# RETRACTION: A Meta‐Analysis of Postoperative Wound Complications at the Surgical Site in Prostate Cancer Patients Undergoing Robotic Surgery

**DOI:** 10.1111/iwj.70574

**Published:** 2025-04-21

## Abstract

**RETRACTION:** SuX. , DaiZ. , ZhaoH. , JiM. , and LiuZ. , “A Meta‐Analysis of Postoperative Wound Complications at the Surgical Site in Prostate Cancer Patients Undergoing Robotic Surgery,” International Wound Journal 21, no. 4 (2024): e14560, 10.1111/iwj.14560.38130091 PMC10961857

The above article, published online on 21 December 2023, in Wiley Online Library (http://onlinelibrary.wiley.com/), has been retracted by agreement between the journal Editor in Chief, Professor Keith Harding; and John Wiley & Sons Ltd. Following an investigation by the publisher, all parties have concluded that this article was accepted solely on the basis of a compromised peer review process. The editors have therefore decided to retract the article. The authors did not respond to our notice regarding the retraction.

**RETRACTION:** X. Su , Z. Dai , H. Zhao , M. Ji , and Z. Liu , “A Meta‐Analysis of Postoperative Wound Complications at the Surgical Site in Prostate Cancer Patients Undergoing Robotic Surgery,” International Wound Journal 21, no. 4 (2024): e14560, 10.1111/iwj.14560.38130091 PMC10961857

The above article, published online on 21 December 2023, in Wiley Online Library (http://onlinelibrary.wiley.com/), has been retracted by agreement between the journal Editor in Chief, Professor Keith Harding; and John Wiley & Sons Ltd. Following an investigation by the publisher, all parties have concluded that this article was accepted solely on the basis of a compromised peer review process. The editors have therefore decided to retract the article. The authors did not respond to our notice regarding the retraction.

